# Cone-Beam Computed Tomography in Orthodontics

**DOI:** 10.3390/dj7030089

**Published:** 2019-09-02

**Authors:** Ahmad Abdelkarim

**Affiliations:** Department of Orthodontics, University of Mississippi Medical Center, 2500 North State Street, Jackson, MS 39216, USA; aabdelkarim@umc.edu

**Keywords:** cone-beam CT, CBCT in orthodontics, CBCT review, orthodontic advanced imaging

## Abstract

Unlike patients receiving implants or endodontic treatment, most orthodontic patients are children who are particularly sensitive to ionizing radiation. Cone-beam computed tomography (CBCT) carries risks and benefits in orthodontics. The principal risks and limitations include ionizing radiation, the presence of artifacts, higher cost, limited accessibility, and the need for additional training. However, this imaging modality has several recognized indications in orthodontics, such as the assessment of impacted and ectopic teeth, assessment of pharyngeal airway, assessment of mini-implant sites, evaluation of craniofacial abnormalities, evaluation of sinus anatomy or pathology, evaluation of root resorption, evaluation of the cortical bone plate, and orthognathic surgery planning and evaluation. CBCT is particularly justified when it brings a benefit to the patient or changes the outcome of the treatment when compared with conventional imaging techniques. Therefore, CBCT should be considered for clinical orthodontics for selected patients. Prescription of CBCT requires judicious and sound clinical judgment. The central question of this narrative review article is: when does CBCT add value to the practice of orthodontics? To answer this question, this article presents discussion on radiation dosage of CBCT and other imaging techniques used in orthodontics, limitations of CBCT in orthodontics, justifying the use of CBCT in orthodontics, and the benefits and evidence-based indications of CBCT in orthodontics. This review summarizes the central themes and topics in the literature regarding CBCT in orthodontics and presents ten orthodontic cases in which CBCT proved to be valuable.

## 1. Introduction

Cone-beam computed tomography (CBCT) is a radiographic technique introduced to the United States dental market in 2001. Since the discovery of the X-ray more than a century ago, few other diagnostic imaging modalities have impacted dental practice to the extent that CBCT has. Since CBCT introduction, the progress made in CBCT maxillofacial applications has been remarkable. 

CBCT technology uses a cone-shaped source of ionizing radiation and a two-dimensional detector [1]. It provides multidimensional and dimensionally accurate images for diagnosis and treatment planning. These images contain isotropic voxels (volume elements) such that each volume element has equal dimensions in all three orthogonal planes, allowing accurate multi-planar images in any direction desired by the practitioner. 

CBCT has attracted significant attention from practitioners who seek to enhance diagnosis and treatment for their patients [2]. Indications of CBCT in orthodontics have been documented. However, risks and limitations of CBCT need to be explored and weighed against the benefits of CBCT in each case. 

Practitioners of the healing arts must minimize harm to their patients. It is therefore necessary to find valid and robust evidence on which to base the selection of CBCT imaging for the orthodontic patient. There is a vast literature on CBCT in orthodontics, including several opposing views. Therefore, the objective of this narrative review is to answer this question: when does CBCT add value to the practice of orthodontics? To answer this question, this article presents discussion on radiation dosage of CBCT in orthodontics, limitations of CBCT in orthodontics, justifying the use of CBCT in orthodontics, and the benefits and evidence-based indications of CBCT in orthodontics. Ten orthodontic cases in which CBCT was utilized are presented. Understanding the indications for CBCT in orthodontics and weighing its risks and benefits allow the orthodontist to be able to prescribe CBCT when it brings value to the orthodontic patient. 

## 2. Radiation Dosage of CBCT in Orthodontics

Theoretically, any amount of ionizing radiation, no matter how small, has the potential to cause a deleterious effect [3]. Radiation is a carcinogen, and current radiation protection protocols are based upon the linear non-threshold (LNT) assumption that even very low doses of radiation can cause cancer. 

Most patients who undergo orthodontic therapy are children [4], and children of orthodontic age are radiosensitive and susceptible to the untoward effects of ionizing radiation [5,6], whereas adults are more resistant. Children have higher risk from ionizing radiation for two reasons: they have higher cell and tissue sensitivity to radiation than adults, and they have a longer lifespan than adults in which radiation-induced changes may manifest [7,8,9,10,11,12].

Radiation carcinogenesis has a stochastic effect which means that the probability of cancer increases with increased dose, but the severity of cancer is not related to the dose [3]. For instance, a similar malignancy developed later in life can be caused by any radiation dosage, but the chance of its occurrence increases with a higher dose. Generally, children’s exposure to low radiation doses has the effect of a small yet insignificant increase in the risk of a fatal cancer that may develop during life [13,14]. 

In addition to the age factor, the risk of cancer arising from radiation varies depending on gender, exposure type (acute or chronic), and radiation type. For instance, female patients are slightly more radiosenstive than male patients [15]. In other words, not all radiation exposures have the same effect. 

There are differences between dental and medical radiographic imaging. In the medical field, medical computed tomography (CT) scans carry the highest risk, and the risk assessment has shown that these scans have become a leading source of future risk to the general population [16]. To estimate the risk of ionizing radiation, the effective dose concept is used. To compute the effective dose, the total amount of absorbed dosages by the tissues is multiplied by the tissue weighting factors [17]. 

CBCT effective doses are smaller than those of medical CT [18,19,20,21,22,23,24,25,26,27]. However, there is a wide range of effective doses that are present across different CBCT machines. This large range of effective doses is strongly correlated with the size of the field of view (FOV) [28]. If the FOV of CBCT is increased, the effective dose increases as well [29]. Reducing the size of the FOV is therefore one of the greatest and easiest ways to reduce the effective dose of CBCT. 

In addition, reducing the scan time, number of projections, and the mAs (Milliampere-seconds) has an additional role in reducing the dose as well [30,31]. In fact, small and strategic adjustments in exposure parameters can result in significant reduction in the effective dose without significantly compromising the image quality [32,33]. However, significant modifications of these parameters aimed at significant reduction of the effective dose can reduce the image quality [28,34]. Therefore, clinical judgment should be exercised with dose reduction efforts in order to maintain diagnostic and quality images. 

Because the dose received is strongly related to the field size, a small FOV can be selected for the region of interest that triggers the interest in CBCT acquisition [35]. In order to optimize the use of CBCT, the FOV should be justifiable, patient-specific, and indication-oriented [36]. An impacted canine, for example, would not require a large volume CBCT scan. A small CBCT volume of 40 × 40 may be sufficient, patient-specific, and indication-oriented. 

Using smaller volumes benefits the patient because it can reduce the effective dose [28,37,38,39,40]. In addition, it benefits the practitioner, because small CBCT volumes do not include areas in the head that are difficult for most dental practitioners to interpret, and thus reduces time spent on radiographic interpretation [41]. 

Whereas effective doses of CBCT are less than those of medical CT, CBCT dosages are generally higher than effective doses of panoramic and cephalometric imaging. The effective dose of a digital panoramic radiograph has the range of 6–38 microSieverts (µSv) [29,42,43,44,45,46,47], and the effective dose of a cephalometric radiograph has the range of 2–10 µSv [23,46,48]. On the other hand, the range of effective doses of CBCT is very large and has been reported to be 5.3–1025 µSv, depending on the size of the FOV, specific technique factors, and the machine itself [25,29,34,37,42,43,44,45,46,47,49,50,51,52,53,54]. One legacy CBCT machine had a large field-of-view setting in which the effective dose exceeded 1000 µSv [52]. To put this in perspective, the effective dose of a medical CT for the head is approximately 1000–2000 µSv [26]. 

It must be stated that most of the current CBCT dosages are in the lower half of the reported range, and significant efforts are being made to standardize different CBCT scanners and to further reduce CBCT dosages to the point that they are close to the panoramic and cephalometric radiographic dosages [55]. As Table 1 demonstrates, the combined panoramic and cephalometric radiographic dosages and the lowest CBCT dosage for some machines and significantly reduced exposure settings (i.e., FOV, mAs, scan time) may actually overlap. Some CBCT machines have the capability of reducing the amount of radiation dose for different patient sizes while maintaining optimal image detail and quality. Moreover, a new technology called the Dose Reduction Technology (DRT) can allow the clinician to set the machine in the DRT mode, which results in low dosages that rival two-dimensional imaging such as panoramic radiography. 

Besides the large range of CBCT reported doses, these values may in fact differ across different ages. For instance, children have higher effective doses because they are smaller than adults [56]. The difference in size between children and adults results in the higher proximity of radiosensitive organs (e.g., thyroid gland) in children to the FOV, which results in a larger effective dose for children [46]. This occurs even if the exposure protocols are exactly the same. Therefore, the cancer risk per unit of radiation dose is higher for children than for adults [57].

Collective effective dose, measured in person-Sv, is another concept in radiation biology. It is defined as the product of the effective dose and the number of individuals exposed. This concept is frequently mentioned in medical imaging because CT scans have high dosages. The collective medical effective dose in the United States of a population of about 300 million was estimated to be 900,000 person-Sv in 2006. This figure is about seven-fold the estimate made in 1982 (124,000 person-Sv), due to the increased popularity of CT scans and nuclear medicine [58]. These two modalities account for 75% of the collective medical effective dose [59], and it is estimated that approximately 1.5 to 2% of all the cancers developing in the U.S. are due to the use of CT alone [60]. 

This may be a public health issue, but it is related mainly to CT scans. As previously mentioned, and as Table 1 demonstrates, CBCT radiation doses are fortunately lower than the corresponding doses for medical CT. Yet there is one resemblance that can be observed here; the increased popularity of CBCT in orthodontics over time will inevitably result in the increase of collective effective dose for orthodontic patients, thus increasing the likelihood of radiation risks in these patients [61,62]. Because children are sensitive to radiation, the use of thyroid protection (lead apron with collar) has been recommended [63]. Lead shielding significantly reduces the effective dose, and is generally an effective way to reduce the risks of ionizing radiation [53]. 

## 3. Limitations and Liability Associated with the Use of CBCT in Orthodontics

Besides the exposure to ionizing radiation, CBCT comes with other limitations and concerns. For example, CBCT scanners have higher cost and limited accessibility when compared to conventional radiographic imaging techniques. In addition, CBCT images are sufficient for visualization of teeth and bone, but are unable to represent the internal structure of soft tissues or soft tissue lesions with high accuracy [64,65]. 

Inherent artifacts that may be present in CBCT images include beam hardening [66]. In general, metal artifacts are observed on CBCT images in the vicinity of metals [67]. In orthodontics, these artifacts can be noted on the images around orthodontic brackets and bands (scattering) [68]. 

Also, CBCT images can display noise, cupping artifacts, or scatter [69]. It is possible to acquire CBCT during orthodontic treatment, but the images may include beam hardening and scatter around orthodontic appliances. Other limitations may include motion artifacts, especially in young orthodontic patients who are more likely to move during long CBCT scans [70]. These limitations inherent to CBCT should be considered because they can affect the image quality. 

CBCT image quality is not comparable across different scanners [71]. There are approximately 50 commercially available CBCT models and scanners with variable image quality. Clinicians who are unfamiliar with CBCT image quality may not be able to compare different scanners in regard to their images. 

While CBCT images are considered accurate and reliable in terms of linear measurements [72,73,74,75,76,77,78], CBCT images may occasionally present false positives and false negatives. For example, CBCT images may not produce a reliable presentation of a thin cortical bone [79]. Misinterpretation of CBCT images may affect orthodontic decision making. Further, an artifact may be confused with the presence of pathology and may therefore lead to false diagnoses. 

Presentation of CBCT images through volume rendering or Maximum Intensity Projection (MIP) may increase the likelihood of false findings. These illustrations are created based on sophisticated software algorithms, and therefore they may not always be accurate. Therefore, evaluation of the volume through axial, sagittal, and coronal views is required. Such evaluation is technically demanding and may be difficult initially for some practitioners. Interpretation of CBCT scans requires skills and knowledge beyond that obtained at dental school [80,81]. 

Finally, with the use of CBCT scanning, the orthodontist bears legal responsibility to report any pathology in the scan [82,83]. There has been significant controversy regarding the orthodontist’s liability to report any pathology evident in the scan. As with any radiographic interpretation, the orthodontist is responsible for interpretation of the CBCT volume in its entirety [84]. In some countries, such as the United States, the full interpretation of CBCT is a legal requirement [82,83,85,86,87]. Some clinicians may choose to refer to an oral and maxillofacial radiologist to transfer these risks [88], and at the same time provide their patients with a specialty level care for the radiographic interpretation of their CBCT scans [89]. 

When several of these risks and limitations inherent to CBCT imaging are mitigated or eliminated, CBCT becomes an excellent tool to enhance orthodontic diagnosis and treatment planning [89], however, the use of CBCT must be justified according to established guidelines. 

## 4. Justifying the Use of CBCT in Orthodontics According to Established Guidelines

In orthodontics, the same set of radiographs should not be routinely made for all patients [90,91]. Orthodontists find the panoramic and cephalometric radiography to be sufficient for most initial, progress, and final records [87,92]. However, CBCT may prove to be advantageous in some clinical encounters. The great advantage of CBCT is that it provides images of various dental, oral, and maxillofacial structures in multiple orthogonal images (i.e., coronal, sagittal, axial). CBCT can also provide curved or flat slices of variable thickness. In addition, CBCT provides multi-planar reformatted images, volume rendering, maximum intensity projection, and other 3D visual representations. 

Orthodontists and dental practitioners should carefully consider any radiographic examination before it is ordered. This process is called image selection or the use of selection criteria. The selection of CBCT in general is based on the patient’s presentation and the need to diagnose, monitor, or evaluate the outcome of a treatment [93]. 

For any case, the orthodontist should be able to justify the use of CBCT. CBCT can be justified if conventional imaging techniques such as panoramic and cephalometric radiographs fail to provide correct diagnosis or when CBCT has a positive effect on treatment options or treatment optimization [94,95]. It need not be considered a standard method of diagnosis in orthodontics because conventional two-dimensional radiographic techniques (e.g., panoramic and cephalometric radiographs) usually suffice for orthodontic diagnosis and treatment planning. 

Because the concerns about radiation risks are heightened for children, who comprise most orthodontic patients, several position statements have been made by respected organizations. Position statements and clinical guidelines made by reputable international health care organizations are authoritative and defensible. They are released after exhaustive review and appraisal of the literature. The Swiss Association of Dentomaxillofacial Radiology recommends that CBCT in orthodontics be used only if it brings additional information compared to conventional two-dimensional imaging [96]. The DIMITRA (Dentomaxillofacial paediatric imaging: an investigation towards low-dose radiation induced risks), a European multicenter and a multidisciplinary project, released a position statement encouraging practitioners to follow the principle of ALADAIP—keeping radiation As Low as Diagnostically Acceptable being Indication-oriented and Patient-specific [36]. The clinically relevant ALADAIP directive is especially relevant for young orthodontic patients. 

Not a single organization recommended CBCT for all orthodontic patients. For example, the American Dental Association recommended that CBCT be prescribed only when there is an expected diagnostic benefit for the patient or significant improvement in the clinical outcome [93]. The American Academy of Oral and Maxillofacial Radiology recommended the use of CBCT imaging in orthodontics only when there is justification made on an individual basis according to the clinical presentation [85]. The British Orthodontic Society guidelines are comparable, and did not recommend CBCT imaging for all orthodontic patients [10]. Therefore, the strongest theme in these recommendations regarding prescription of CBCT in orthodontics is that CBCT must be justified on a case-by-case basis and when it has the potential to improve diagnosis or treatment. Prescribing CBCT for all orthodontic patients may be considered a flawed and questionable practice [97]. 

Despite robust justification of CBCT in selected cases, some authors found insignificant differences in treatment planning decisions when CBCT was used versus conventional imaging [98], and others have stated that, even though CBCT may alter treatment planning, it does not necessarily improve or change orthodontic treatment outcome [99,100,101]. It is difficult to assess the exact value of CBCT with regards to changing the orthodontic treatment outcome because the evidence on CBCT efficacy and diagnostic value is not obtained from randomized controlled trials, but rather mostly from observational studies or studies with variable hierarchy of evidence [102,103]. 

## 5. Benefits and Evidence-Based Indications of CBCT in Orthodontics

CBCT brings specific and unique diagnostic benefits in orthodontics [104]. The most common indication for CBCT in orthodontics is the 3D assessment of anomalies in dental position such as impactions and ectopic teeth [94,105,106,107,108,109]. CBCT allows the visualization of impacted teeth in three dimensions, as well as the evaluation of roots of the impacted and adjacent teeth. 

It has been suggested that in cases with impacted maxillary canines, CBCT can actually alter treatment planning decisions [107,110,111,112,113]. This is due to the fact that conventional panoramic or intraoral radiography may not provide a good assessment of the root status of adjacent teeth, but with CBCT this can be done effectively [114,115]. This is especially true in cases with severe displacement of the impacted tooth in which an accurate assessment of the impacted and adjacent teeth is essential [116,117,118]. Justification of CBCT in these cases increases given that CBCT brings significant value to diagnosis and treatment planning. 

In addition to the assessment of anomalies in dental position, CBCT provides information on the stage of dental development, and position and size of the tooth or follicle [119]. CBCT can also provide a great tool for evaluation and detection of any supernumerary teeth [120]. 

Patients with dentofacial abnormalities and deformities can benefit from CBCT [109]. For example, CBCT can be prescribed for patients with facial asymmetry, cleft palate, or obstructive sleep apnea [94,109,121,122,123,124,125,126,127]. Because structures such as cleft palate and oropharyngeal airway are three-dimensional, it is advantageous to use CBCT for the evaluation of these structures [109,128]. CBCT also provides three-dimensional assessment for alveolar boundary conditions, craniofacial anatomy, and maxillary transverse dimensions [129]. CBCT can be used in craniofacial orthodontics in which effects of maxillary expansion, evaluation of the clefts, and the skeletal and soft tissues can be assessed in all dimensions [130,131]. Incidental findings or pathologies discovered via 2D imaging, such as panoramic radiograph, can be better visualized via CBCT. This is especially valuable if the orthodontist desires to evaluate the pathology in three-dimensions and its relationship to the teeth. 

If temporary anchorage devices such as mini-implants or mini-plates are planned before or during orthodontic treatment, CBCT can help the practitioner in evaluating the proposed site for insertion or the status of the temporary anchorage device after the insertion [132,133,134,135,136,137,138,139,140,141,142,143]. 

If the evaluation of the temporomandibular joints (TMJs) is required, CBCT has the potential to provide information about the bony component of the TMJs [144,145,146,147]. CBCT provides better evaluation of the shape and volume of the TMJ condyles when compared to panoramic radiography [94]. However, the articular disk and muscles cannot be visualized via CBCT [70,148]. These structures are well visualized through magnetic resonance imaging (MRI). 

Unlike 2D superimpositions provided by conventional cephalometric radiography, CBCT can provide the clinician with sophisticated 3D superimpositions and treatment assessment when necessary [149,150,151,152,153]. Assessment of orthognathic surgery can be made via these superimpositions [154,155]. In addition, assessment of soft tissue changes of the face in orthognathic surgery cases can be made [156,157]. Whereas CBCT can be used for evaluation of orthodontic surgical cases, the use of CBCT in these cases does not necessarily alter treatment outcome [65]. 

One of the great features of CBCT is its ability to construct different views, such as a panoramic view of the teeth and adjacent structures and another cephalometric view. Therefore, if a large volume CBCT is made, these views can be generally made without taking additional 2D panoramic and cephalometric radiographs. These images can be reconstructed from the CBCT volume, provided that it includes all areas of interest. Several studies confirmed that the cephalometric view synthesized from CBCT volume is equivalent to the conventional cephalometric radiograph in terms of landmark identification, cephalometric analyses, and the overall diagnostic value [158,159,160,161,162,163,164]. 

Unlike conventional panoramic imaging (commonly known as the panorex image), CBCT synthesized panoramic views have the advantage of eliminating magnification, ghost images, distortion, and overlaps. However, creating a panoramic view from the CBCT volume should be made with caution in order to obtain a proper and reliable image [165]. The focal trough can be controlled with CBCT synthesized panoramic radiography, whereby it can be modified and customized to the individual’s jaw size. For example, it can be increased in the anterior region if the patient has bimaxillary dentoalveolar protrusion, or it can be modified in shape if any impacted or ectopic teeth are present. This results in visualization of objects that would otherwise be located outside the focal trough in conventional panoramic radiography. Finally, the size of the focal trough itself can be decreased or increased. For example, if a practitioner uses a focal trough of 20 mm in width for most cases, the focal trough can be increased to 30 mm in a case of bimaxillary dentoalveolar protrusion in which the teeth are proclined. The ability to change the size of the focal trough in this case results in inclusion of the full length of both maxillary and mandibular incisors in the focal trough. 

## 6. Following the ALARA and ALADAIP Principles

Practitioners should always follow the basic ALARA directive in radiation protection, keeping radiation “As Low As Reasonably Achievable [166].” A more evolved and specific directive in radiation protection is the ALADAIP principle [36]. It requires practitioners to keep radiation As Low As Diagnostically Acceptable being Indication-oriented and Patient-specific. 

The ubiquitous and erroneous practice of taking a large volume CBCT for the whole head merely to synthesize panoramic and cephalometric views does not follow the ALADAIP directive, because it does not keep radiation as low as diagnostically acceptable, and it is neither indication-oriented nor patient-specific. If the orthodontic patient requires only two-dimensional panoramic and cephalometric radiographs, these radiographs could be taken without the additional exposure burden that comes with large CBCT volumes [166,167,168]. It also behooves the practitioner to utilize all 3D capabilities of the CBCT scan, and not to be limited to the two-dimensional panoramic and cephalometric views if a large volume is taken. 

Whereas panoramic and cephalometric radiographs may not suffice for specific diagnostic tasks, intraoral radiography may be considered in lieu of CBCT imaging. For example, periapical radiographs may suffice for specific diagnostic tasks, such as assessment of root shapes or root resorption or fracture [169,170] or the evaluation of periodontal status [171]. In other words, if panoramic and cephalometric radiographs are insufficient for these diagnostic tasks, the orthodontist could consider periapical radiography instead of considering CBCT. 

When all conventional radiographic techniques are insufficient for diagnosis and treatment, and the orthodontic patient will benefit from CBCT, the clinician should not hesitate to order this imaging technique. If there is a diagnostic benefit to the patient from CBCT in terms of diagnosis and treatment planning, then this benefit outweighs the risks involved [172,173]. Some patients can benefit dramatically from images provided by CBCT [174]. Therefore, the orthodontist should not hesitate to order a CBCT scan if certain diagnostic information is needed, particularly if this information cannot be obtained via conventional imaging. However, the scan should always be customized to the patient’s needs whenever possible, including the customization of the FOV and other exposure settings in order to reduce and optimize the patient’s ionizing radiation exposure [175,176]. 

## 7. Case Series

The following orthodontic cases provide examples where CBCT was used for diagnosis and treatment planning to obtain information not possible through conventional 2D imaging.

**Figure 1:**
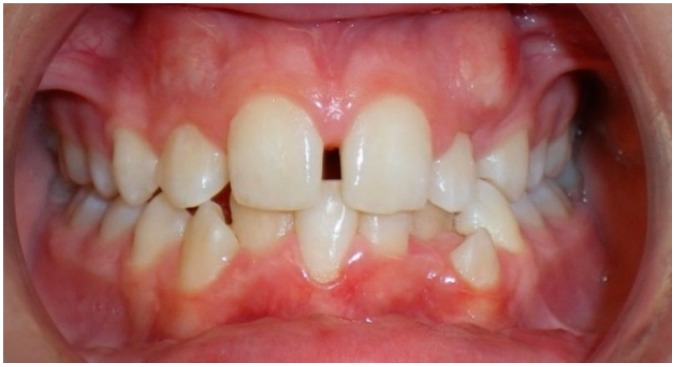
Intraoral photograph of a case with impacted maxillary canines.

**Figure 2:**
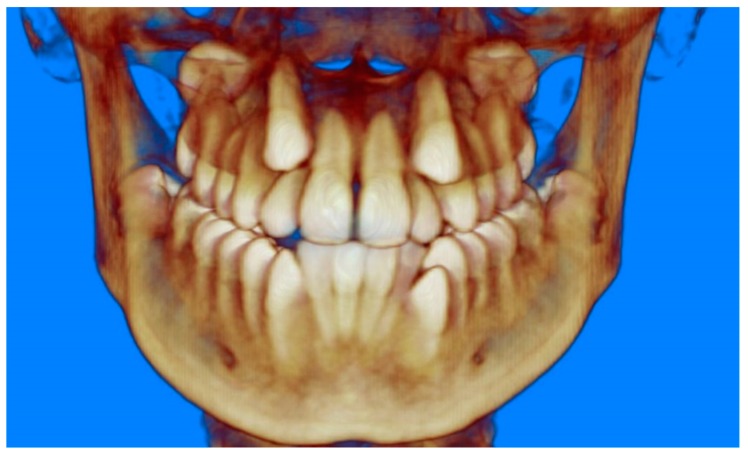
CBCT volume rendering.

**Figure 3:**
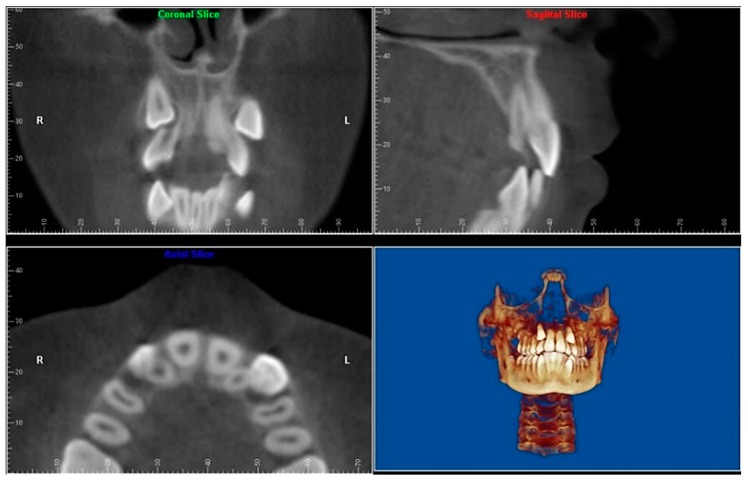
Coronal, sagittal, axial and volume rendering views.

**Figure 4:**
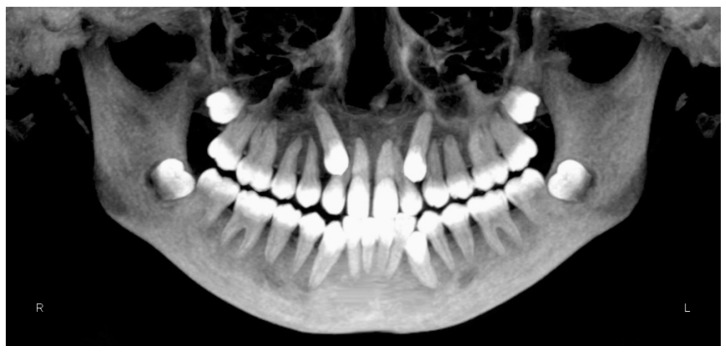
Maximum intensity projection.

**Figure 5:**
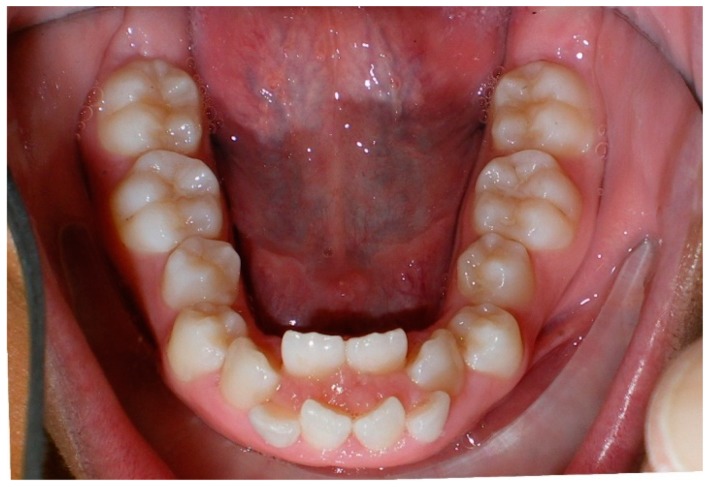
Crowding of mandibular anterior teeth.

**Figure 6:**
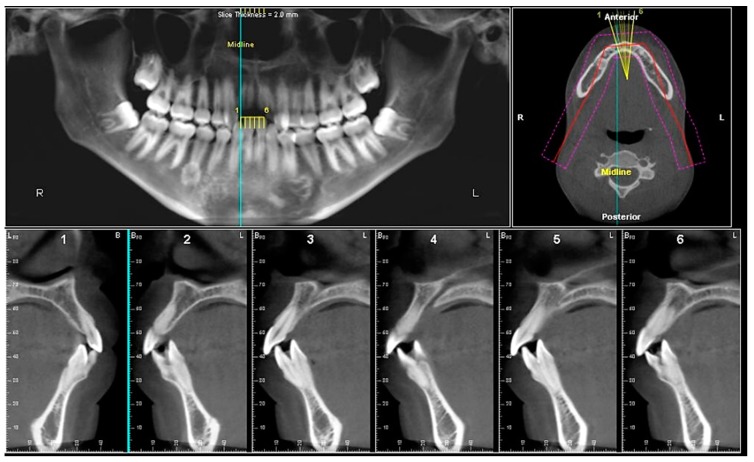
Panoramic, axial, and six cross-sectional views.

**Figure 7:**
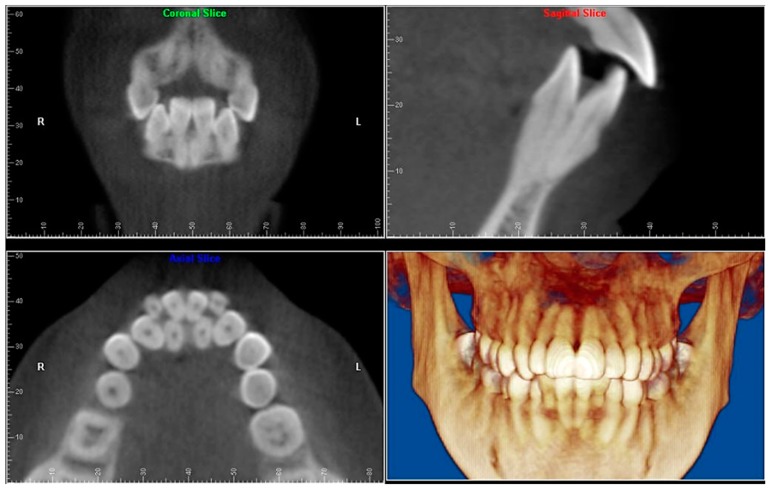
Coronal, sagittal, axial views, and volume rendering views.

**Figure 8:**
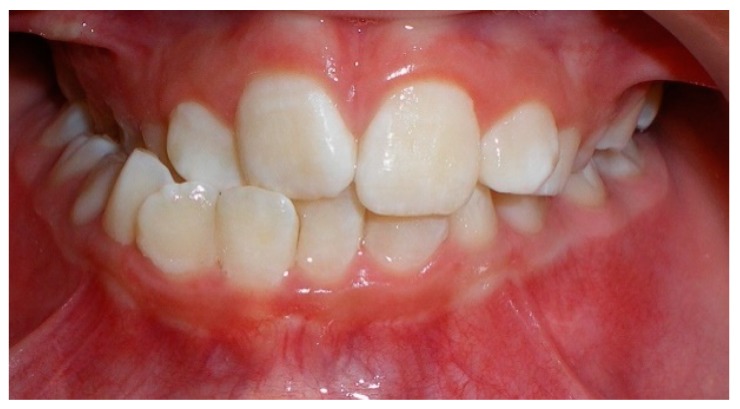
Intraoral photograph showing unilateral posterior crossbite on right side.

**Figure 9:**
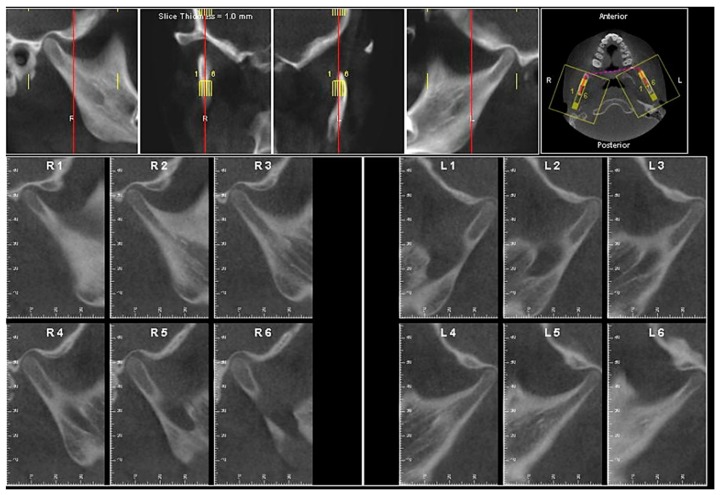
Cross-sectional views of the right and left temporomandibular joints (TMJs).

**Figure 10:**
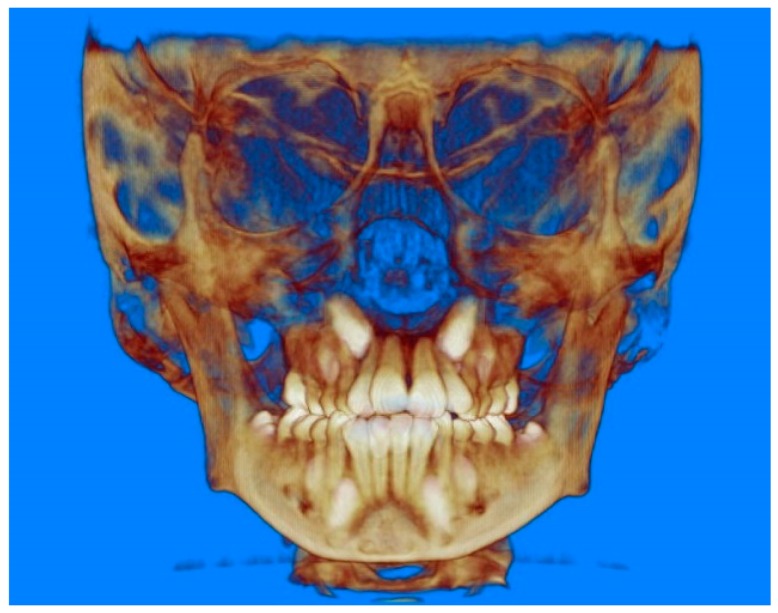
Volume rendering of the CBCT volume.

**Figure 11:**
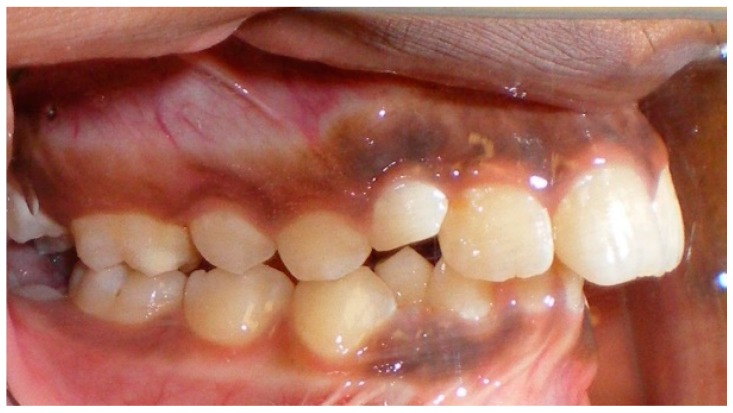
Pre-treatment intraoral photograph.

**Figure 12:**
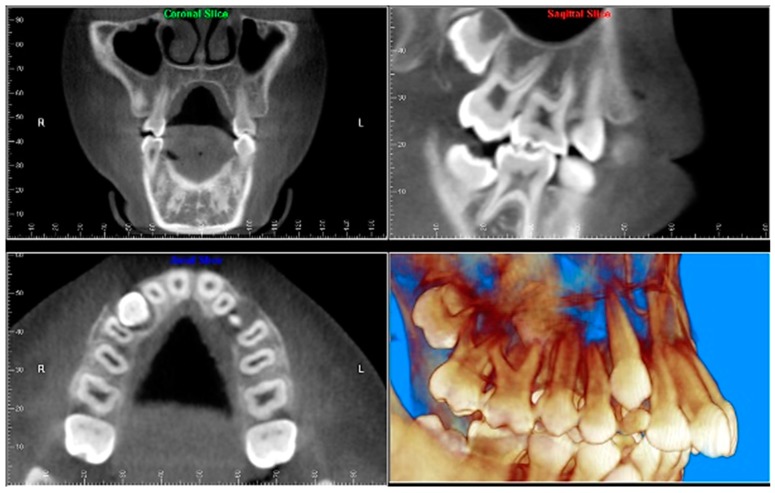
Multiple CBCT views.

**Figure 13:**
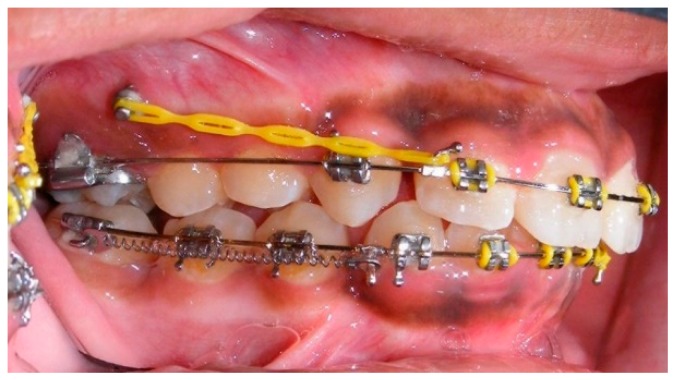
Temporary anchorage device.

**Figure 14:**
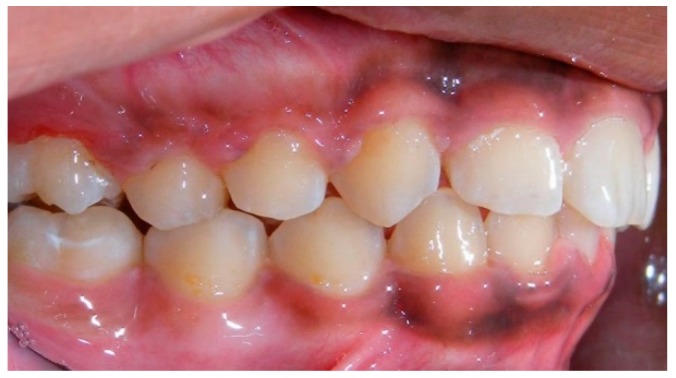
Post-treatment intraoral photograph.

**Figure 15:**
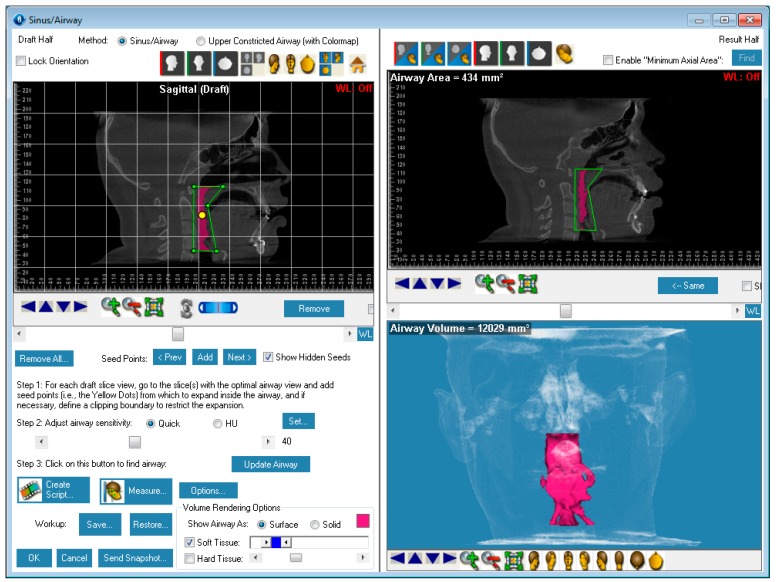
Example of a measurement of the oropharyngeal airway volume and area.

**Figure 16:**
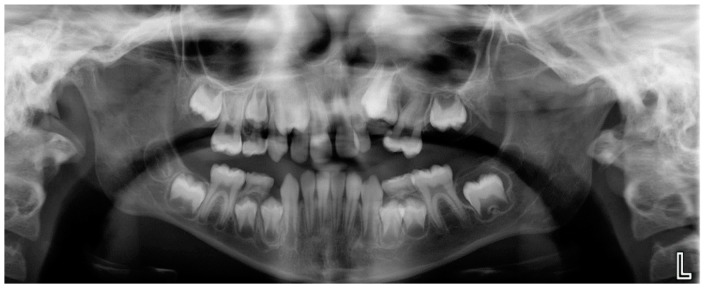
A conventional 2D panoramic radiograph not depicting the ankylosed and submerged primary maxillary left second molar.

**Figure 17:**
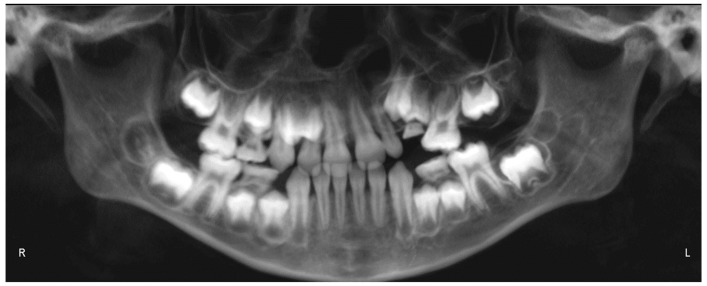
A panoramic view derived from CBCT volume depicting the ankylosed and submerged primary maxillary left second molar.

**Figure 18:**
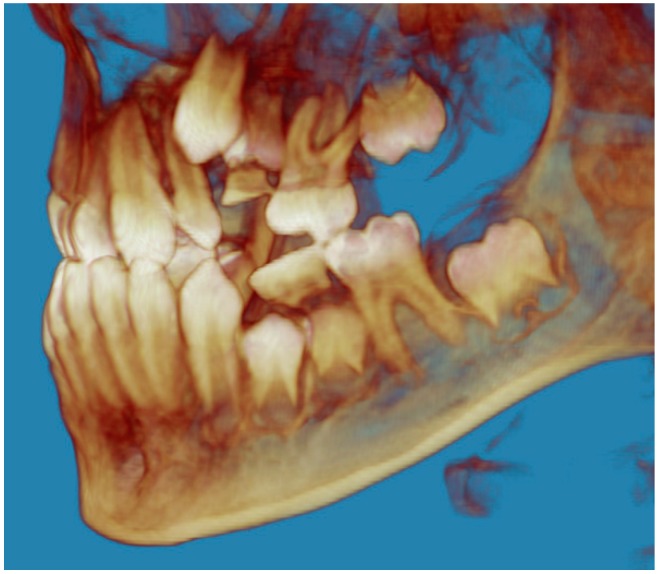
After tooth ankylosis and submerge.

**Figure 19:**
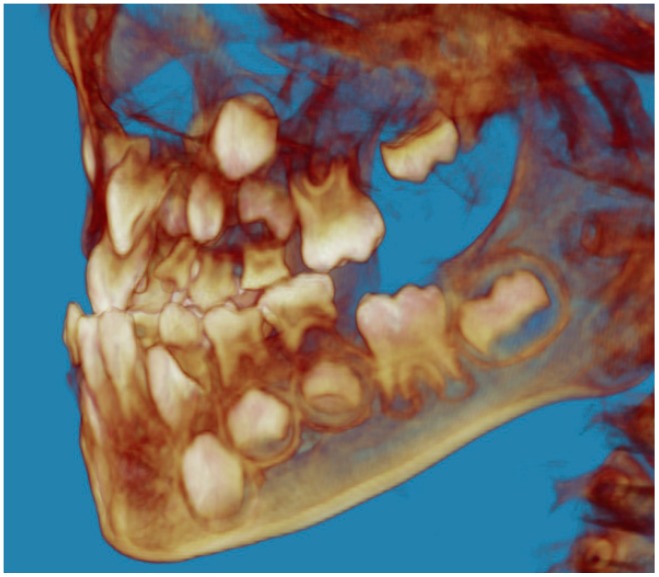
Before tooth ankylosis and submerge.

**Figure 20:**
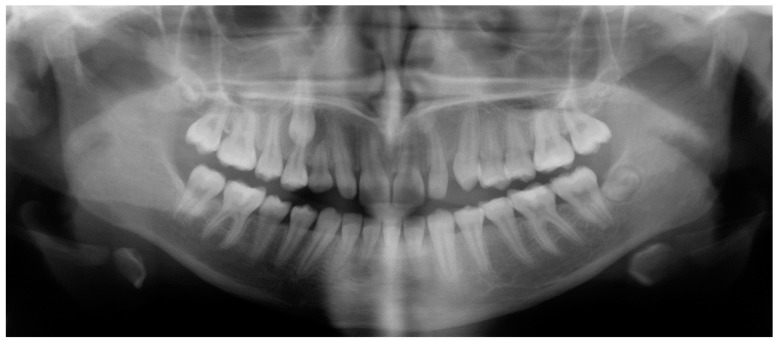
A conventional 2D panoramic radiograph that did not depict accurate status of the canine and first premolar.

**Figure 21:**
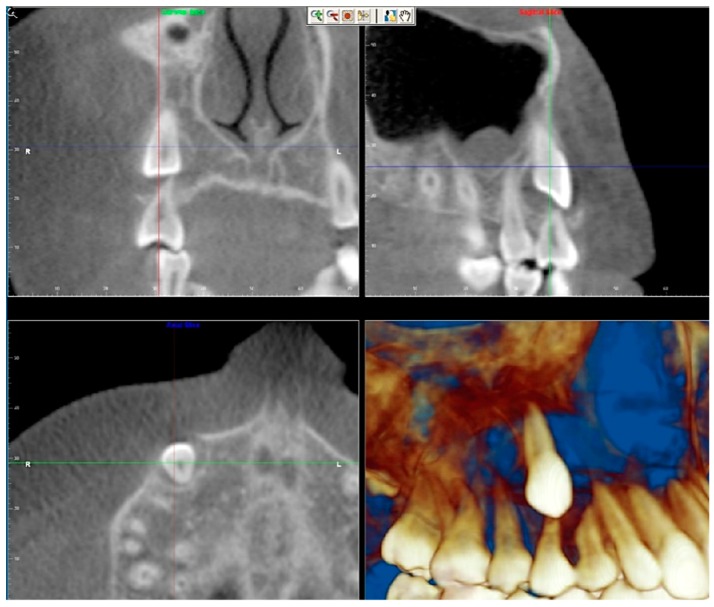
Coronal, sagittal, axial views, and volume rendering showing the impacted canine and its relationship to adjacent structures.

**Figure 22:**
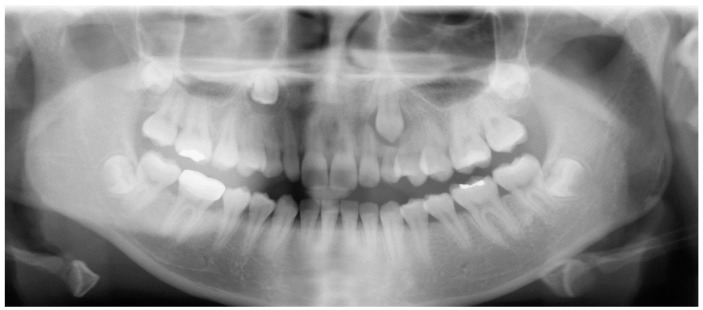
A conventional 2D panoramic radiograph represents limited information on the permanent maxillary right canine.

**Figure 23:**
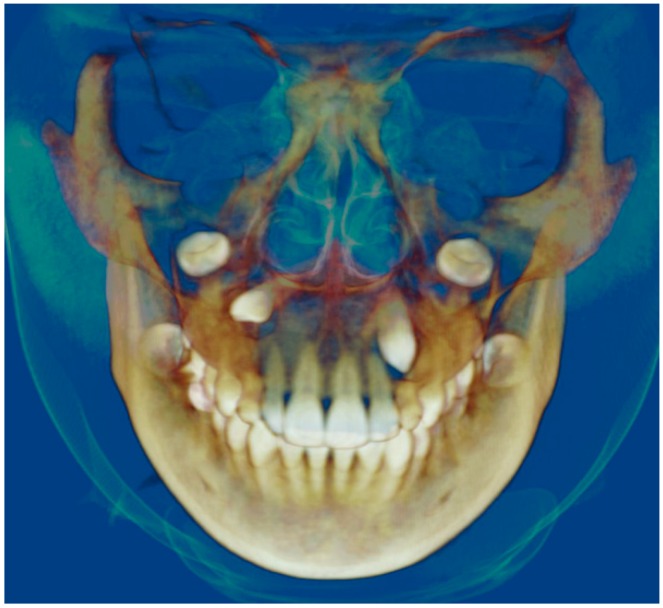
Volume rendering of CBCT.

**Figure 24:**
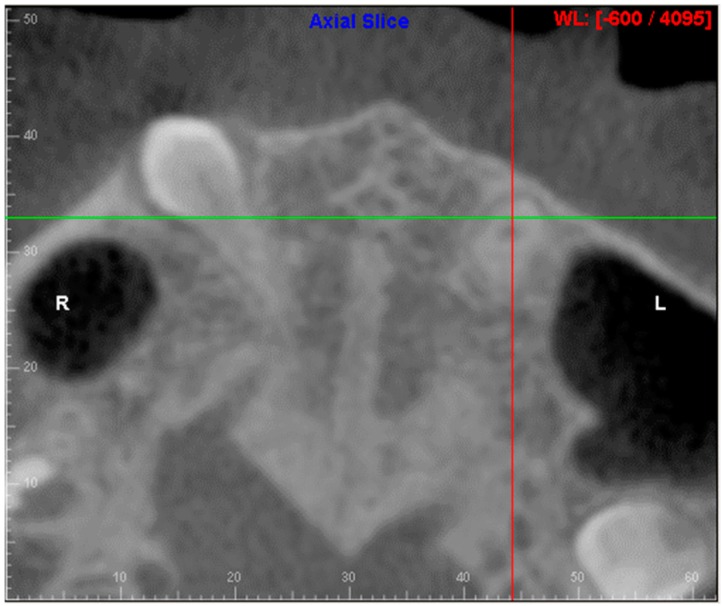
Axial view at the level of the impacted canine.

**Figure 25:**
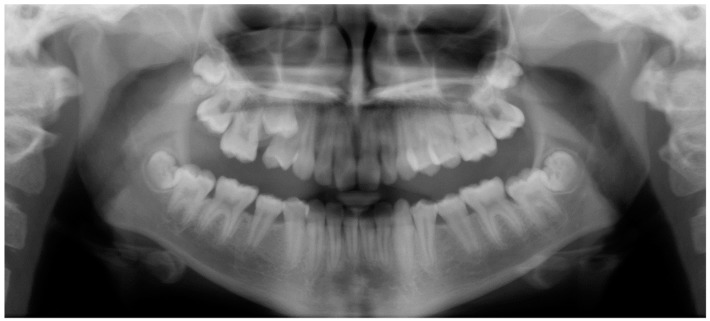
A conventional 2D panoramic radiograph showing limited information about the location of the impacted maxillary right second premolar.

**Figure 26:**
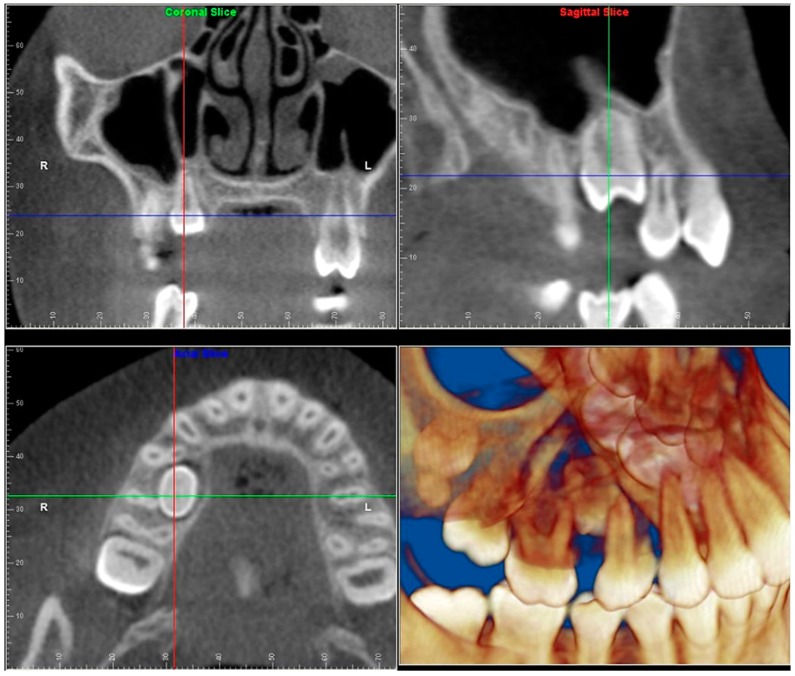
Coronal, sagittal, axial views, and volume rending, showing significant information about location of the impacted premolar.

**Figure 27:**
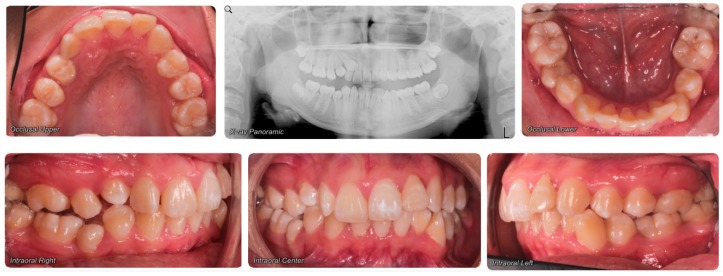
Photographs and 2D panoramic radiograph of a case with an impacted maxillary right canine.

**Figure 28:**
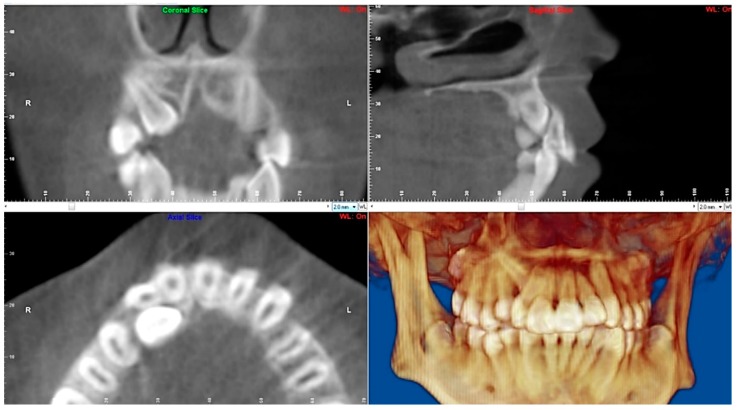
Coronal, sagittal, axial views, and volume rending, showing significant information about the location of the impacted maxillary right canine.

**Figure 29:**
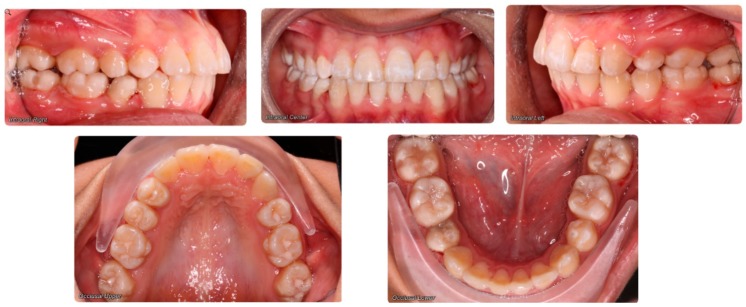
Photographs taken after completion of orthodontic treatment.

**Figure 30:**
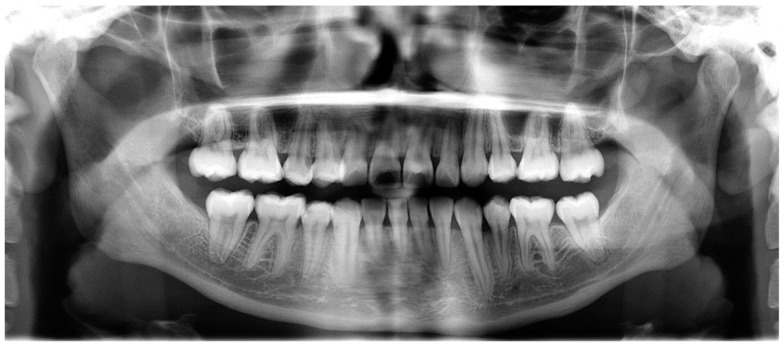
Post-treatment panoramic radiograph.

1. Evaluation of impacted teeth, a common indication of CBCT in orthodontics. The advantages of CBCT include assessment of the tooth location and position, the stage of development, and status of adjacent teeth. CBCT is justified in these cases, because CBCT has the capability of evaluating the impacted teeth and adjacent structures more accurately than 2D conventional imaging. The benefit–risk ratio is favorable, especially if the CBCT volume is collimated to the impacted tooth. Figure 1, Figure 2, Figure 3 and Figure 4 show an example of impacted maxillary canines, and their proximity to the maxillary lateral incisors. Figure 1 shows an intraoral photograph. The benefit of CBCT acquisition in this case includes the ability to visualize the canines and the lateral incisors in three dimensions, which can be visualized in Figure 2 and Figure 3. In this case, the maxillary right lateral incisor exhibited external root resorption, a finding that would be difficult to see on a conventional 2D panoramic radiograph. Figure 4 shows a Maximum Intensity Projection of a panoramic view derived from the CBCT volume. This unique view is free of magnification, distortion, ghost images, and overlaps frequently seen in conventional 2D panoramic radiography.
2. Evaluation of buccal and lingual cortical plates: Figure 5, Figure 6 and Figure 7 show a case in which the mandibular lateral incisors are positioned lingual to the central incisors. Both mandibular lateral incisors are adjacent to each other. Figure 5 shows and intraoral occlusal photos with retained deciduous mandibular lateral incisors. There was no way to evaluate the buccal and lingual cortical plates through conventional 2D panoramic, periapical or occlusal radiographs. Therefore, CBCT was acquired and collimated to the area of teeth in order to assess the relationship of the four mandibular incisors to the labial and lingual cortical plates as well as to the adjacent teeth. As Figure 6 and Figure 7 display, CBCT shows that all permanent mandibular incisors are sound. It is important to note that thin buccal and lingual cortical plates may not be seen via CBCT—this does not denote that they are not present. In other words, CBCT images may not show a clinically present thin buccal and lingual cortical plates. In this case, the diagnostic information obtained from CBCT is far more significant than the information obtained from any other radiographic imaging technique.
3. TMJ and facial asymmetry evaluation. Figure 8, Figure 9 and Figure 10 show a case in which a whole head CBCT was acquired initially due to the presence of facial asymmetry and history of temporomandibular disorders. Figure 8 shows an intraoral photograph with a unilateral posterior crossbite on the right side, a mandibular midline shift to the right side, and an anterior crossbite on the right lateral incisors. Figure 9 shows cross-sectional views of the TMJ, with a very mild flattening of the joints. Figure 10 shows volume rendering of the CBCT volume, demonstrating lack of symmetry of the face, unilateral posterior crossbite observed on the right side involving premolars and molars, and ectopic canines. The benefits of CBCT imaging in this case are the evaluation the TMJ, visualization of the crossbite on the right side via the volume rendering view, and the ability to perform any isometric measurements, if needed.
4. Assessment of proposed sites of temporary anchorage device (TAD). Figure 11, Figure 12, Figure 13 and Figure 14 show correction of the Class II molar relationship using a temporary anchorage device. Figure 11 shows a pre-treatment intraoral photograph of the right side. The Class II molar relationship can be observed. Figure 12 shows coronal, sagittal and axial views, as well as a volume rendering of CBCT that was acquired in order to assess the site of the temporary anchorage device. Figure 13 shows an intraoral photograph of the right side, in which the TAD was placed mesial to the maxillary first molar, and a power chain was attached from this TAD to a hook placed distal to the lateral incisor. Figure 14 shows a post-treatment intraoral photograph showing improvement of the Class II molar relationship after removal of all orthodontic appliances.
5. Oropharyngeal airway assessment. In the past, airway assessment was made using conventional 2D cephalometric radiographs. However, the airway is a three-dimensional structure; it is thus best imaged by a three-dimensional imaging technique. The benefit of CBCT in airway studies is the ability to measure the volume size and evaluate the airway in three dimensions. This is valuable for diagnosis and treatment planning in several cases, especially orthognathic surgery cases. Using CBCT volume, it is possible to measure oropharyngeal airway volume and area. Figure 15 shows a measurement of oropharyngeal airway volume and area via Dolphin 3D Imaging software version 11.95 (Dolphin Imaging and Management Solutions, Chatsworth, CA, USA).
6. Assessment of an ankylosed and submerged primary tooth. Due to limitations of panoramic radiography, objects located outside of the focal trough may not be well visualized. In addition, it may be difficult in some cases to visualize objects that are located within the focal trough. Figure 16 presents an example of a conventional 2D panoramic radiograph in which it was impossible to visualize an ankylosed and submerged primary maxillary left second molar for a child who was 11 years of age. There are two findings that can be seen on the conventional panoramic radiograph: a transposition between the maxillary right canine and first premolar, and a missing maxillary left first premolar. However, the impacted primary molar in the upper left quadrant is not depicted on the conventional panoramic radiograph in Figure 16. After acquisition of CBCT, which was made on the same day the 2D panoramic radiograph was taken, it was possible to see the primary tooth. Figure 17 shows a panoramic view derived from the CBCT volume which shows the ankylosed and submerged primary maxillary left second molar. This tooth can also be seen in the CBCT volume rendering in Figure 18. Interestingly, the patient had another CBCT scan taken approximately three years earlier when the child was 8 years of age. The earlier scan explained the etiology for the problems in the upper left quadrant. The earlier CBCT, displayed in Figure 19, shows that the primary maxillary left second molar was fully erupted and present in the mouth. After the primary tooth became ankylosed, it gradually became severely infraoccluded and then became completely submerged. Meanwhile, the adjacent permanent maxillary left first molar drifted mesially due to lack of space mesial to the tooth, and at the same time the ankylosed primary molar obstructed the eruption of its succedaneous premolar.
7. Assessment of an impacted maxillary canine located superior to a first premolar. Occasionally, transposed or impacted teeth are seen in unusual positions which require accurate diagnosis and treatment planning. Figure 20 presents a 2D conventional panoramic radiograph in which the permanent maxillary right canine can be seen in an unusual position. CBCT was prescribed in order to assess the location of the canine, its relationship to adjacent structures, and the status of the first premolar root. Figure 21 shows CBCT views of the impacted canine and its close proximity to the root of the first premolar. In addition, external root resorption on the first premolar can be visualized. An oral and maxillofacial pathologist evaluated the pericoronal radiolucency adjacent to the crown of the canine, ruled out cystic transformation, and confirmed that it was a hyperplastic follicle. Because the apex of the canine is distal to the apex of the first premolar, coupled with the unusual position of the canine, the orthodontist decided in this case to first extract the primary maxillary right canine, mesially move the maxillary right first premolar to the site of the canine, and then simply extrude the canine via orthodontic traction and place it in the site of the first premolar.
8. Assessment of a horizontally impacted maxillary canine. Figure 22, Figure 23 and Figure 24 show a case in which the permanent maxillary right canine was impacted in a horizontal position. As Figure 22 shows, the conventional 2D panoramic radiograph does not depict the accurate position of the maxillary right canine. On the other hand, it shows some information about the location and status of development of the permanent maxillary left canine. For instance, extraction of the primary maxillary left canine could be followed by orthodontic traction of the succedaneous tooth. However, this would not be realistic for the right canine. As Figure 23 and Figure 24 show, the right canine is impacted in a horizontal position. The apex of this canine is in close proximity to the right nasal fossa. An attempt to bring this tooth into alignment would carry significant risks. For example, the tooth may be ankylosed, its movement may damage adjacent teeth or structures, it may become devitalized or infected, and most importantly, it can result in a significantly prolonged orthodontic treatment. Orthodontic movement of this canine would likely be ruled out by most orthodontists. The patient’s parents can either choose to extract this tooth or monitor it long term. A referral to an oral and maxillofacial surgeon can be valuable in order to discuss options for management of this impacted tooth. The CBCT volume can be of significant value for the oral and maxillofacial surgeon for locating and evaluating the tooth accurately, after which the surgeon can present to the patient’s parents the risks and benefits of extracting the tooth versus leaving it and monitoring its status long term.
9. Assessment of an impacted maxillary premolar. Figure 25 and Figure 26 show a case in which the permanent maxillary right second premolar was rotated and impacted in an unusual position. As Figure 25 shows, it is impossible to accurately evaluate the position of the impacted premolar from the conventional 2D panoramic radiograph. Three-dimensional evaluation of the impacted tooth is necessary. To visualize the tooth in three dimensions, CBCT was acquired. Figure 26 shows a coronal, sagittal, and axial views of the impacted premolar, as well as a volume rending. It can be noted that the impacted premolar is rotated in a pattern in which the buccal cusp is in the vicinity of the first premolar and the lingual cusp is in the vicinity of the first molar. In addition, the impacted tooth is in a palatal position. The orthodontic treatment plan included leveling and aligning, followed by opening space for this tooth and then bringing it to the dental arch via orthodontic traction. CBCT images provided in Figure 26 are valuable for orthodontic diagnosis and treatment plan, and would also be valuable for the surgeon who will perform the surgical exposure of the tooth and bonding of a gold chain which will be used to extrude the impacted premolar.
10. Assessment of an impacted canine with close proximity to the lateral incisor. Figure 27 shows photographs and a panoramic radiograph of a case in which there is an impacted permanent maxillary right canine in an unfavorable position, a missing mandibular left second premolar and uncoordinated dental midlines. The relationship of the impacted canine to the adjacent lateral incisor cannot be determined from the conventional 2D radiograph. Therefore, CBCT was acquired. Figure 28 shows CBCT images, including coronal, sagittal, axial views, and volume rendering, which demonstrated close proximity of the impacted canine to the lateral incisor, and an area of bone loss buccal to the crown of the impacted canine. Before acquisition of CBCT, the tentative treatment plan was to extract the maxillary right first premolar and bring the canine to the dental arch. However, due to the findings presented by CBCT, the treatment plan was altered in favor of extracting the impacted canine, a clinical decision that was strongly favored by the patient. In this case, the first premolar would substitute for the canine. The maxillary left first premolar and mandibular right first premolar were also extracted. Therefore, each quadrant would have one missing tooth by end of treatment. Orthodontic post-treatment photographs are presented in Figure 29. Figure 30 shows a post-treatment 2D panoramic radiograph. CBCT was neither necessary nor indicated at completion of orthodontic treatment, and therefore only a conventional 2D panoramic radiograph was taken.

## 8. Conclusions

Some orthodontic patients can benefit from CBCT’s capability to improve diagnosis and treatment planning. Appropriate use of CBCT by acquiring CBCT only when necessary has the potential to reduce ionizing radiation exposure to orthodontic patients. Generally, the risks of CBCT in orthodontics are outweighed by the benefits that CBCT scans provide in selected cases in which conventional radiographs cannot provide sufficient information necessary for diagnosis and treatment planning. 

There is a strong consensus amongst position statements released by international organizations regarding CBCT in orthodontics, stating that CBCT is justified only when it brings a benefit to the patient or changes the outcome of the orthodontic treatment when compared with conventional imaging techniques. In these selected cases, the recommendation is to use the smallest possible FOV, with the lowest radiation exposure. 

Therefore, CBCT can provide orthodontists with valuable diagnostic information, but its use should be case specific in which the clinician should be able to justify the reason for CBCT acquisition. Prescribing CBCT regularly for all patients increases the collective dose for orthodontic patients and is not consistent with international guidelines for an appropriate use of ionizing radiation in orthodontics. Consequently, CBCT in orthodontics requires judicious and sound clinical judgement. 

## Figures and Tables

**Table 1:** Comparison between the effective dose of digital panoramic radiography, cephalometric radiography, cone-beam computed tomography (CBCT), and medical computed tomography (CT).

Imaging Technique	Range of Effective Dose (µSv) Reported in the Literature
Panoramic radiography	6–38
Cephalometric radiography	2–10
CBCT	5.3–1025
Medical head CT	1000–2000

## Abbreviations

CBCT	cone-beam computed tomography
FOV	field of view
µSv	microSieverts
TMJ	temporomandibular joint
